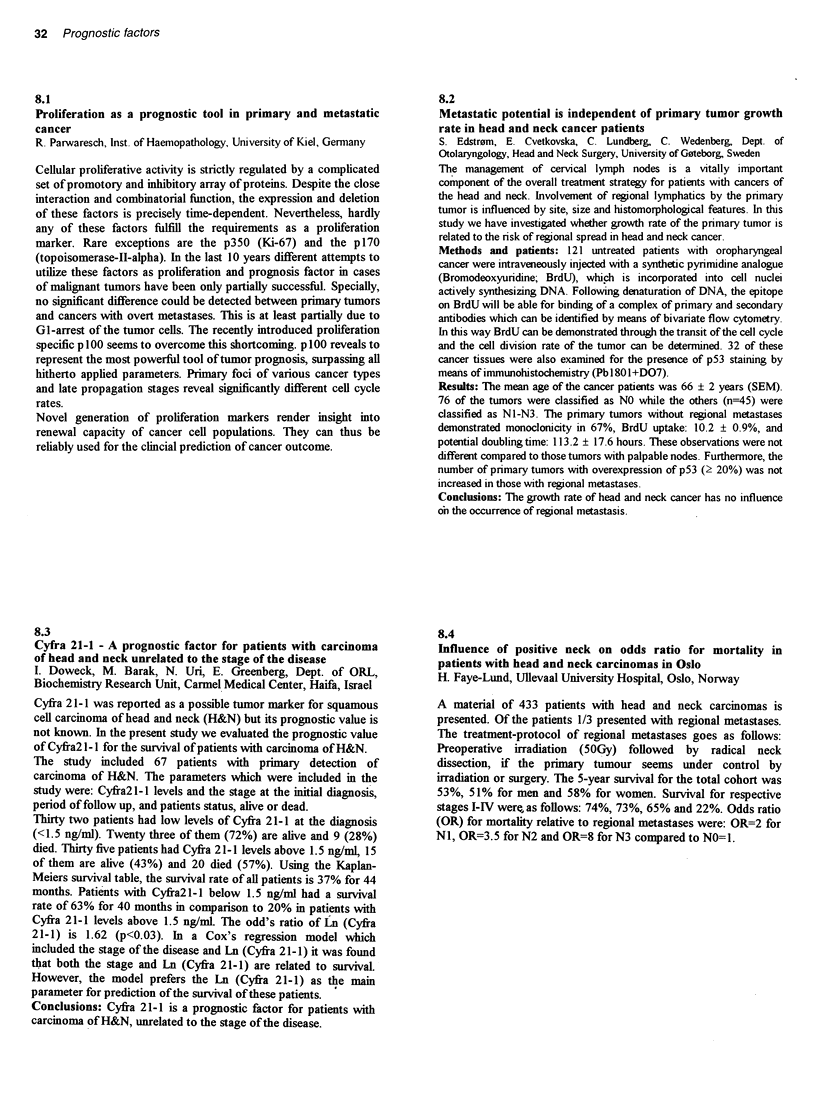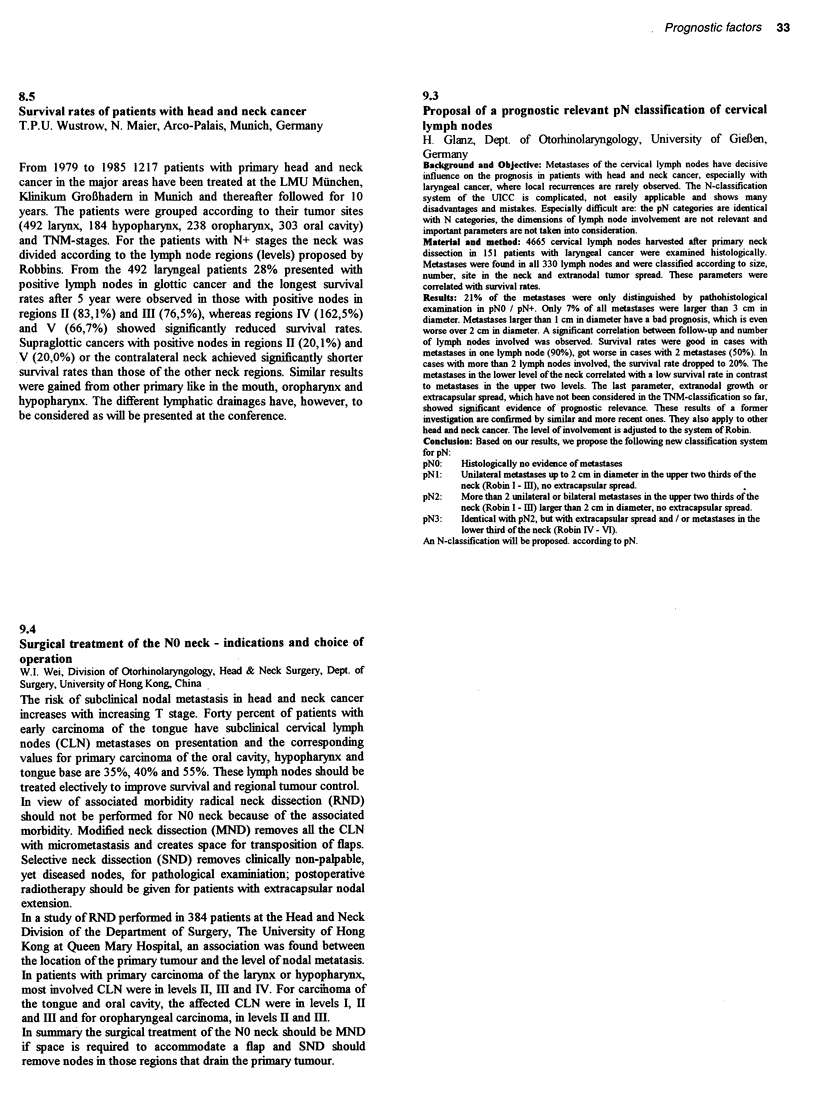# Prognostic factors

**Published:** 1998

**Authors:** 


### 8.1
### Proliferation as a prognostic tool in primary and metastatic cancer
R. Parwaresch, Inst. of Haemopathology, University of Kiel, Germany

Cellular proliferative activity is strictly regulated by a complicated set of promotory and inhibitory array of proteins. Despite the close interaction and combinatorial function, the expression and deletion of these factors is precisely time-dependent. Nevertheless, hardly any of these factors fulfill the requirements as a proliferation marker. Rare exceptions are the p350 (Ki-67) and the p170 (topoisomerase-II-alpha). In the last 10 years different attempts to utilize these factors as proliferation and prognosis factor in cases of malignant tumors have been only partially successful. Specially, no significant difference could be detected between primary tumors and cancers with overt metastases. This is at least partially due to G1-arrest of the tumor cells. The recently introduced proliferation specific p100 seems to overcome this shortcoming. p100 reveals to represent the most powerful tool of tumor prognosis, surpassing all hitherto applied parameters. Primary foci of various cancer types and late propagation stages reveal significantly different cell cycle rates.
Novel generation of proliferation markers render insight into renewal capacity of cancer cell populations. They can thus be reliably used for the clincial prediction of cancer outcome.

### 8.2
### Metastatic potential is independent of primary tumor growth rate in head and neck cancer patients
S. Edstrøm, E. Cvetkovska, C. Lundberg, C. Wedenberg, Dept. of Otolaryngology, Head and Neck Surgery, University of Gøteborg, Sweden

The management of cervical lymph nodes is a vitally important component of the overall treatment strategy for patients with cancers of the head and neck. Involvement of regional lymphatics by the primary tumor is influenced by site, size and histomorphological features. In this study we have investigated whether growth rate of the primary tumor is related to the risk of regional spread in head and neck cancer.
**Methods and patients:** 121 untreated patients with oropharyngeal cancer were intraveneously injected with a synthetic pyrimidine analogue (Bromodeoxyuridine; BrdU), which is incorporated into cell nuclei actively synthesizing DNA. Following denaturation of DNA, the epitope on BrdU will be able for binding of a complex of primary and secondary antibodies which can be identified by means of bivariate flow cytometry. In this way BrdU can be demonstrated through the transit of the cell cycle and the cell division rate of the tumor can be determined. 32 of these cancer tissues were also examined for the presence of p53 staining by means of immunohistochemistry (Pb1801+DO7).
**Results:** The mean age of the cancer patients was 66 ± 2 years (SEM). 76 of the tumors were classified as N0 while the others (n=45) were classified as N1-N3. The primary tumors without regional metastases demonstrated monoclonicity in 67%, BrdU uptake: 10.2 ± 0.9%, and potential doubling time: 113.2 ± 17.6 hours. These observations were not different compared to those tumors with palpable nodes. Furthermore, the number of primary tumors with overexpression of p53 (≥ 20%) was not increased in those with regional metastases.
**Conclusions:** The growth rate of head and neck cancer has no influence on the occurrence of regional metastasis.

### 8.3
### Cyfra 21-1 - A prognostic factor for patients with carcinoma of head and neck unrelated to the stage of the disease
I. Doweck, M. Barak, N. Uri, E. Greenberg, Dept. of ORL, Biochemistry Research Unit, Carmel Medical Center, Haifa, Israel

Cyfra 21-1 was reported as a possible tumor marker for squamous cell carcinoma of head and neck (H&N) but its prognostic value is not known. In the present study we evaluated the prognostic value of Cyfra21-1 for the survival of patients with carcinoma of H&N.
The study included 67 patients with primary detection of carcinoma of H&N. The parameters which were included in the study were: Cyfra21-1 levels and the stage at the initial diagnosis, period of follow up, and patients status, alive or dead.
Thirty two patients had low levels of Cyfra 21-1 at the diagnosis (<1.5 ng/ml). Twenty three of them (72%) are alive and 9 (28%) died. Thirty five patients had Cyfra 21-1 levels above 1.5 ng/ml, 15 of them are alive (43%) and 20 died (57%). Using the Kaplan-Meiers survival table, the survival rate of all patients is 37% for 44 months. Patients with Cyfra21-1 below 1.5 ng/ml had a survival rate of 63% for 40 months in comparison to 20% in patients with Cyfra 21-1 levels above 1.5 ng/ml. The odd's ratio of Ln (Cyfra 21-1) is 1.62 ($p<0.03$). In a Cox's regression model which included the stage of the disease and Ln (Cyfra 21-1) it was found that both the stage and Ln (Cyfra 21-1) are related to survival. However, the model prefers the Ln (Cyfra 21-1) as the main parameter for prediction of the survival of these patients.
**Conclusions:** Cyfra 21-1 is a prognostic factor for patients with carcinoma of H&N, unrelated to the stage of the disease.

### 8.4
### Influence of positive neck on odds ratio for mortality in patients with head and neck carcinomas in Oslo
H. Faye-Lund, Ullevaal University Hospital, Oslo, Norway

A material of 433 patients with head and neck carcinomas is presented. Of the patients 1/3 presented with regional metastases. The treatment-protocol of regional metastases goes as follows: Preoperative irradiation (50Gy) followed by radical neck dissection, if the primary tumour seems under control by irradiation or surgery. The 5-year survival for the total cohort was 53%, 51% for men and 58% for women. Survival for respective stages I-IV were as follows: 74%, 73%, 65% and 22%. Odds ratio (OR) for mortality relative to regional metastases were: OR=2 for N1, OR=3.5 for N2 and OR=8 for N3 compared to N0=1.

### 8.5
### Survival rates of patients with head and neck cancer

T.P.U. Wustrow, N. Maier, Arco-Palais, Munich, Germany

From 1979 to 1985 1217 patients with primary head and neck cancer in the major areas have been treated at the LMU München, Klinikum Großhadern in Munich and thereafter followed for 10 years. The patients were grouped according to their tumor sites (492 larynx, 184 hypopharynx, 238 oropharynx, 303 oral cavity) and TNM-stages. For the patients with N+ stages the neck was divided according to the lymph node regions (levels) proposed by Robbins. From the 492 laryngeal patients 28% presented with positive lymph nodes in glottic cancer and the longest survival rates after 5 year were observed in those with positive nodes in regions II (83,1%) and III (76,5%), whereas regions IV (162,5%) and V (66,7%) showed significantly reduced survival rates. Supraglottic cancers with positive nodes in regions II (20,1%) and V (20,0%) or the contralateral neck achieved significantly shorter survival rates than those of the other neck regions. Similar results were gained from other primary like in the mouth, oropharynx and hypopharynx. The different lymphatic drainages have, however, to be considered as will be presented at the conference.

### 9.4
### Surgical treatment of the N0 neck - indications and choice of operation

W.I. Wei, Division of Otorhinolaryngology, Head & Neck Surgery, Dept. of Surgery, University of Hong Kong, China

The risk of subclinical nodal metastasis in head and neck cancer increases with increasing T stage. Forty percent of patients with early carcinoma of the tongue have subclinical cervical lymph nodes (CLN) metastases on presentation and the corresponding values for primary carcinoma of the oral cavity, hypopharynx and tongue base are 35%, 40% and 55%. These lymph nodes should be treated electively to improve survival and regional tumour control.

In view of associated morbidity radical neck dissection (RND) should not be performed for N0 neck because of the associated morbidity. Modified neck dissection (MND) removes all the CLN with micrometastasis and creates space for transposition of flaps. Selective neck dissection (SND) removes clinically non-palpable, yet diseased nodes, for pathological examiniation; postoperative radiotherapy should be given for patients with extracapsular nodal extension.

In a study of RND performed in 384 patients at the Head and Neck Division of the Department of Surgery, The University of Hong Kong at Queen Mary Hospital, an association was found between the location of the primary tumour and the level of nodal metatasis. In patients with primary carcinoma of the larynx or hypopharynx, most involved CLN were in levels II, III and IV. For carcinoma of the tongue and oral cavity, the affected CLN were in levels I, II and III and for oropharyngeal carcinoma, in levels II and III.

In summary the surgical treatment of the N0 neck should be MND if space is required to accommodate a flap and SND should remove nodes in those regions that drain the primary tumour.

### 9.3
### Proposal of a prognostic relevant pN classification of cervical lymph nodes

H. Glanz, Dept. of Otorhinolaryngology, University of Gießen, Germany

**Background and Objective:** Metastases of the cervical lymph nodes have decisive influence on the prognosis in patients with head and neck cancer, especially with laryngeal cancer, where local recurrences are rarely observed. The N-classification system of the UICC is complicated, not easily applicable and shows many disadvantages and mistakes. Especially difficult are: the pN categories are identical with N categories, the dimensions of lymph node involvement are not relevant and important parameters are not taken into consideration.

**Material and method:** 4665 cervical lymph nodes harvested after primary neck dissection in 151 patients with laryngeal cancer were examined histologically. Metastases were found in all 330 lymph nodes and were classified according to size, number, site in the neck and extranodal tumor spread. These parameters were correlated with survival rates.

**Results:** 21% of the metastases were only distinguished by pathohistological examination in pN0 / pN+. Only 7% of all metastases were larger than 3 cm in diameter. Metastases larger than 1 cm in diameter have a bad prognosis, which is even worse over 2 cm in diameter. A significant correlation between follow-up and number of lymph nodes involved was observed. Survival rates were good in cases with metastases in one lymph node (90%), got worse in cases with 2 metastases (50%). In cases with more than 2 lymph nodes involved, the survival rate dropped to 20%. The metastases in the lower level of the neck correlated with a low survival rate in contrast to metastases in the upper two levels. The last parameter, extranodal growth or extracapsular spread, which have not been considered in the TNM-classification so far, showed significant evidence of prognostic relevance. These results of a former investigation are confirmed by similar and more recent ones. They also apply to other head and neck cancer. The level of involvement is adjusted to the system of Robin.

**Conclusion:** Based on our results, we propose the following new classification system for pN:

pN0: Histologically no evidence of metastases pN1: Unilateral metastases up to 2 cm in diameter in the upper two thirds of the neck (Robin I - III), no extracapsular spread.

pN2: More than 2 unilateral or bilateral metastases in the upper two thirds of the neck (Robin I - III) larger than 2 cm in diameter, no extracapsular spread.

pN3: Identical with pN2, but with extracapsular spread and / or metastases in the lower third of the neck (Robin IV - VI).

An N-classification will be proposed. according to pN.